# Cold Atmospheric Plasma Potentiates the Photodynamic Effects of Protoporphyrin IX-Loaded Mesoporous Silica-Coated Iron Oxide Nanoclusters in HaCaT Cells

**DOI:** 10.3390/nano16161012

**Published:** 2026-08-17

**Authors:** Demet Erdag, Harun Basoglu, Leman Yalcintepe, Muhammet S. Toprak

**Affiliations:** 1 Department of Computer Programming, Vocational School, Biruni University, 34015 Istanbul, Türkiye; derdag@kth.se; 2 Department of Applied Physics, KTH Royal Institute of Technology, SE-106 91 Stockholm, Sweden; 3 Department of Biophysics, Faculty of Medicine, Bilecik Seyh Edebali University, 11100 Bilecik, Türkiye; 4 Department of Biophysics, Faculty of Medicine, Istanbul University, 34093 Istanbul, Türkiye

**Keywords:** cold atmospheric plasma—CAP, protoporphyrin IX, mesoporous silica-coated iron oxide nanoclusters, photodynamic therapy, apoptosis, reactive oxygen species

## Abstract

Photodynamic therapy (PDT) is a reactive oxygen species (ROS)-based treatment modality whose efficacy is often limited by poor photosensitizer stability and delivery. In this study, mesoporous silica-coated iron oxide nanoclusters (MNCs) were synthesized and loaded with protoporphyrin IX (PPIX) to obtain a multifunctional PPIX@MNC nanoplatform. Physicochemical characterization was performed using transmission electron microscopy (TEM), dynamic light scattering (DLS), zeta potential analysis, and Fourier-transform infrared spectroscopy (FTIR). The biological effects of MNC, free PPIX, and PPIX@MNC were evaluated in HaCaT cells—as a general epithelial model—under dark conditions, light irradiation, cold atmospheric plasma (CAP) exposure, and combined CAP-assisted photodynamic treatment. FTIR, DLS, and zeta potential analyses confirmed successful incorporation of PPIX into the nanoclusters. Cell viability assays revealed pronounced phototoxicity of free PPIX, with the IC50 value decreasing from 44.4 ± 3.5 nM under dark conditions to 14 ± 2 nM following light activation, corresponding to a phototoxicity index of 3.17. CAP further enhanced PPIX-mediated cytotoxicity, and the CAP-assisted photodynamic group exhibited the strongest response, with an IC50 value of 9.6 ± 1.1 nM. CAP further enhanced PPIX-mediated cytotoxicity. Increased ROS generation, enhanced apoptosis, and marked mitochondrial membrane potential disruption were observed particularly in CAP-Light-PPIX-treated cells. Although encapsulation of PPIX within MNCs reduced acute cytotoxicity compared with free PPIX, the nanoplatform retained responsiveness to light and CAP stimulation. These findings demonstrate that CAP potentiates PPIX-mediated photodynamic effects through enhanced oxidative stress and suggest that mesoporous silica-coated magnetic nanoclusters represent a promising platform for controlled photosensitizer delivery in CAP-assisted PDT applications.

## 1. Introduction

Reactive oxygen and nitrogen species (RONS)-based therapeutic strategies have attracted considerable attention in recent years due to their ability to selectively induce oxidative stress and regulate cellular signaling pathways [1,2,3]. Among these approaches, photodynamic therapy (PDT) represents one of the most extensively investigated modalities for the treatment of various malignant and non-malignant diseases [4,5,6]. Among current therapeutic strategies for cancer treatment, chemotherapy, radiotherapy, sonodynamic therapy (SDT), and photothermal therapy (PTT) have all demonstrated significant therapeutic potential [7]. Chemotherapy remains the standard treatment for many malignancies but is frequently associated with systemic toxicity and the development of drug resistance [8]. Radiotherapy provides effective local tumor control but may also damage surrounding healthy tissues [9]. In contrast, SDT and PTT offer non-invasive alternatives; however, they require specialized energy sources and still face challenges related to treatment selectivity and penetration depth [7]. Compared with these approaches, photodynamic therapy (PDT) offers unique advantages, including precise spatial and temporal control of ROS generation, minimal invasiveness, reduced systemic toxicity, and the possibility of repeated treatment [10]. PDT relies on the activation of a photosensitizer by light of an appropriate wavelength, resulting in the generation of reactive oxygen species (ROS), particularly singlet oxygen (^1^O_2_), which subsequently induces oxidative damage to cellular biomolecules and triggers cell death pathways [4,11].

Cancer remains one of the leading causes of morbidity and mortality worldwide. According to GLOBOCAN 2022, approximately 20.0 million new cancer cases and 9.7 million cancer-related deaths occurred globally, emphasizing the urgent need for more effective and selective therapeutic strategies [12]. Beyond oncology, PDT has also been successfully employed for several non-malignant disorders, including actinic keratosis, Bowen’s disease, acne vulgaris, and certain chronic infectious diseases, with actinic keratosis representing one of the most common clinical indications for PDT in dermatology [13,14,15]. Despite its clinical success, the therapeutic efficacy of PDT remains restricted by insufficient photosensitizer accumulation, poor aqueous solubility, aggregation-induced quenching, off-target toxicity and limited light penetration [5,16], thereby motivating the development of nanocarrier-based platforms and combination approaches such as cold atmospheric plasma-assisted PDT.

Protoporphyrin IX (PPIX) is a naturally occurring porphyrin and one of the most widely studied photosensitizers due to its strong photoactivity and ability to generate singlet oxygen following light activation [17,18]. Upon light irradiation, PPIX efficiently transfers absorbed energy to molecular oxygen, producing ROS capable of damaging cellular membranes, proteins, and nucleic acids [17,19]. However, free PPIX suffers from poor stability, aggregation tendency, and limited cellular delivery, which may reduce its therapeutic performance and increase nonspecific toxicity [20,21]. Therefore, the development of nanocarrier systems capable of improving PPIX delivery while minimizing adverse effects has become an important research focus [5,16,22].

Nanotechnology-based delivery systems offer several advantages for PDT applications, including enhanced photosensitizer loading, improved physicochemical stability, reduced aggregation/self-quenching, and increased cellular uptake [23,24]. Recent advances in nanocarrier design have demonstrated that rationally designed drug delivery systems can ensure drug stability, bioavailability, targeted delivery, and controlled release, further highlighting their ability to enhance therapeutic efficacy while minimizing off-target effects [25]. Mesoporous silica nanoclusters (MSNs) have attracted particular attention because of their high surface area, tunable pore structure, favorable biocompatibility, and ease of surface functionalization [24,26]. Furthermore, the incorporation of iron oxide magnetic nanocores into mesoporous silica structures enables the fabrication of multifunctional nanoplatforms with improved physicochemical properties and potential theranostic applications [27,28]. Iron oxide nanoparticles have attracted considerable attention as multifunctional nanoplatforms because of their favorable magnetic properties, biocompatibility, imaging capability, and therapeutic potential across a wide range of biomedical applications, including targeted drug delivery and regenerative medicine [29]. The mesoporous silica shell can effectively accommodate photosensitizers such as PPIX and reduce acute toxicity associated with free photosensitizer exposure.

Cold atmospheric plasma (CAP) has emerged as a promising biomedical technology capable of generating a complex mixture of reactive oxygen and nitrogen species, including hydrogen peroxide (H_2_O_2_), hydroxyl radicals (^•^OH), singlet oxygen (^1^O_2_), nitric oxide (NO), and peroxynitrite (ONOO^−^). These reactive species can induce oxidative stress, lipid peroxidation, mitochondrial dysfunction, and apoptosis [1,2,3,30]. In recent years, CAP has been increasingly investigated as an adjuvant strategy capable of enhancing the efficacy of conventional anticancer therapies, including chemotherapy, radiotherapy, and photodynamic therapy [30,31,32]. The synergistic interaction between CAP-generated reactive species and PDT-induced oxidative stress may result in amplified ROS production and improved therapeutic outcomes [31,33].

Several studies have demonstrated that combining CAP with photosensitizers can significantly increase ROS-associated cytotoxic responses compared with either treatment alone. However, the interaction between CAP and PPIX-loaded mesoporous silica-coated iron oxide nanoclusters remains poorly understood. Limited information is available regarding how nanocluster-mediated delivery influences the biological response to CAP-assisted PDT and whether nanocluster loading alters acute cytotoxicity, phototoxicity, and oxidative stress generation.

In the present study, mesoporous silica-coated iron oxide nanoclusters (MNC) were synthesized and loaded with PPIX to obtain a multifunctional PPIX@MNC nanoplatform. The physicochemical properties of the nanoclusters were characterized using transmission electron microscopy (TEM), dynamic light scattering (DLS), zeta potential measurements, and Fourier-transform infrared spectroscopy (FTIR). Subsequently, the effects of free PPIX, MNC, and PPIX@MNC on HaCaT cells were investigated under dark conditions, light irradiation, CAP exposure, and combined CAP-assisted photodynamic treatment. Cell viability, phototoxicity, ROS generation, and apoptosis-related responses were evaluated to determine the contribution of nanocluster loading and CAP treatment to PPIX-mediated biological activity. Furthermore, IC50 values and enhancement indices were calculated to quantitatively assess treatment efficacy and the potential interaction between CAP and photodynamic activation. To the best of our knowledge, this is the first study to investigate the biological effects of protoporphyrin IX-loaded mesoporous silica-coated iron oxide nanoclusters (PPIX@MNC) in combination with cold atmospheric plasma and photodynamic activation in HaCaT cells.

## 2. Materials and Methods

### 2.1. Materials

Iron(III) chloride (FeCl3), ethylene glycol, ethanol, sodium acetate, sodium citrate, tetraethyl orthosilicate (TEOS), cetyltrimethylammonium bromide (CTAB), NaOH, acetonitrile, protoporphyrin IX (PPIX), dimethyl sulfoxide (DMSO), Cell Counting Kit-8 (CCK-8), Annexin V-FITC/PI apoptosis detection kit, and 2′,7′-dichlorofluorescein diacetate (DCFH-DA) were purchased from Merck (Darmstadt, Germany) and used without further purification. HaCaT cells were maintained in DMEM/F-12 (Gibco, Grand Island, NY, USA) medium supplemented with 10% heat-inactivated fetal bovine serum (FCS), L-glutamine, and antibiotics (Gibco, Grand Island, NY, USA). Cell Count Kit-8 (CCK-8) was purchased from Abcam (Cambridge, UK). Cell cultures were maintained in a Heraeus incubator (Hanau, Germany) in a humidified environment containing 5% CO_2_ at 37 °C. All reagents were of analytical grade.

### 2.2. Synthesis of Mesoporous Silica-Coated Iron Oxide Nanoclusters (MNC)

**Figure 1:**
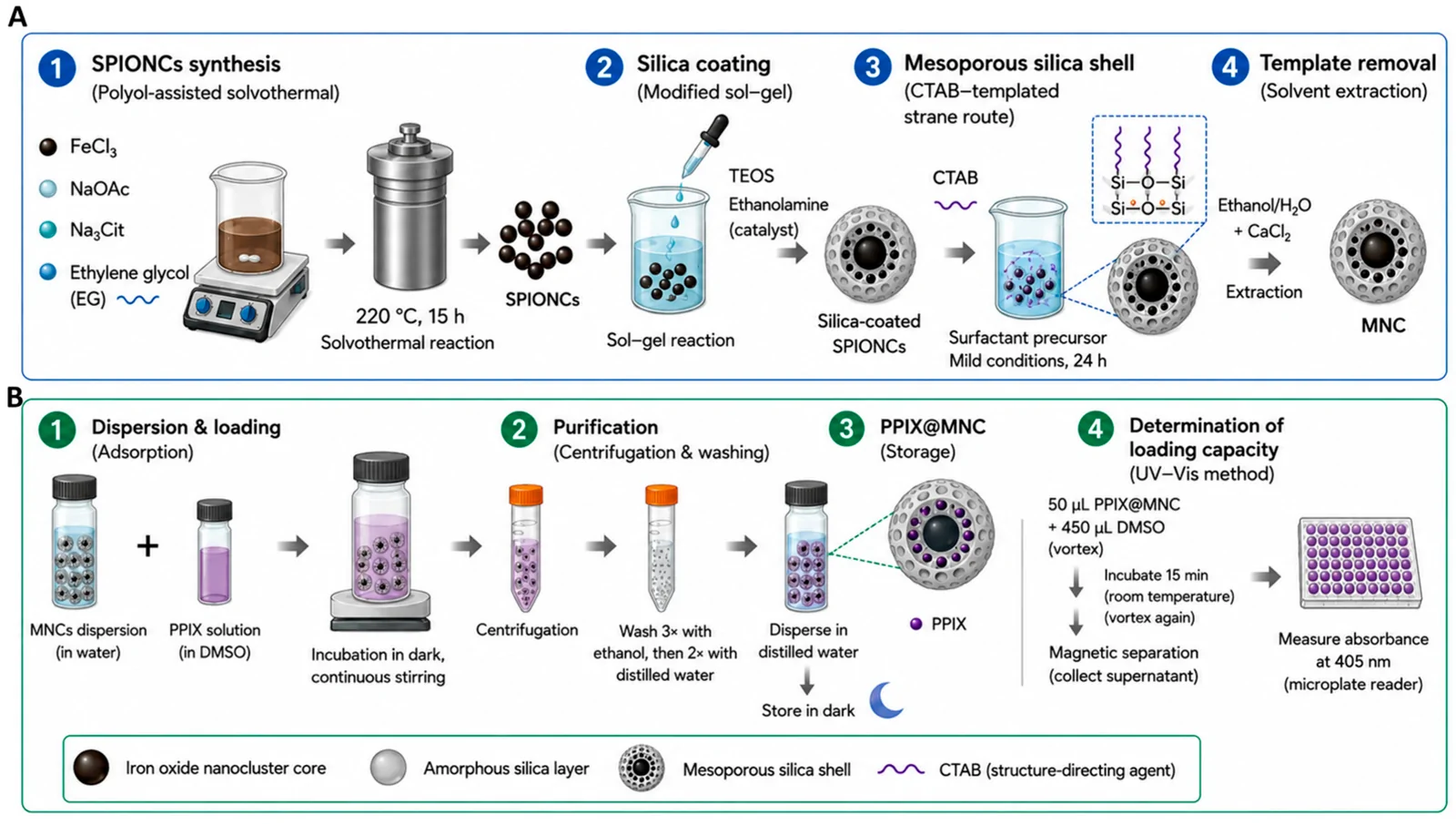
Schematic representation of Synthesis of (**A**) Mesoporous Silica-Coated Iron Oxide Nanoclusters (MNC) and (**B**) Preparation of PPIX-Loaded Nanoclusters (PPIX@MNC).

Superparamagnetic iron oxide nanoclusters (SPIONCs) were synthesized using a polyol-assisted solvothermal method, as previously described [34] and a schematic representation is shown in Figure 1A. Briefly, ferric chloride (FeCl_3_) was used as the iron precursor, sodium acetate (NaOAc) as the alkaline source, ethylene glycol (EG) as both solvent and reducing agent, and trisodium citrate (Na_3_Cit) as the stabilizing agent. The reaction mixture was transferred into a Teflon-lined stainless-steel autoclave and maintained at 220 °C for 15 h to obtain SPIONCs. To improve colloidal stability and prevent nanocluster aggregation, the synthesized SPIONCs were subsequently coated with an amorphous silica layer via a modified sol–gel process. Silica-coated SPIONCs were prepared using TEOS as the silica precursor in the presence of ethanolamine as a catalyst. The resulting nanoclusters were purified by repeated washing with deionized water and ethanol. Mesoporous silica shells were then generated using a CTAB-templated atrane route. Briefly, CTAB was employed as a structure-directing agent, while the silica network was formed using a silatrane precursor under mild reaction conditions [35,36]. Following 24 h of incubation, the obtained particles were collected, purified, and designated as mesoporous silica-coated iron oxide nanoclusters (MNCs). Finally, CTAB was removed by solvent extraction in an ethanol/water mixture containing CaCl_2_, followed by centrifugation, washing, and vacuum drying at 40 °C [24]. The resulting MNCs were used for subsequent loading and biological studies. MNCs, PPIX, and CAP combinations named as described in Table 1.

### 2.3. Preparation of PPIX-Loaded Nanoclusters (PPIX@MNC)

PPIX-loaded nanoclusters (PPIX@MNC) were prepared as follows, MNCs were dispersed in a PPIX solution in DMSO and incubated in dark under continuous stirring to facilitate adsorption of the photosensitizer within the mesoporous structure Figure 1B. PPIX loading onto the nanoclusters was investigated at three different MNC:PPIX mass ratios (20:1, 50:1, and 100:1, *w*/*w*). After loading, the nanoclusters were recovered by centrifugation, washed three times with ethanol and then three times with distilled water to remove unencapsulated PPIX, dispersed in distilled water, and then stored in dark for later use.

The loading capacity of PPIX was determined using a UV-Vis spectrophotometric method. For PPIX extraction, 50 μL of PPIX@MNC suspension was mixed with 450 μL of dimethyl sulfoxide (DMSO) and vortexed to promote the release of PPIX from the nanocluster matrix. The mixture was incubated at room temperature for 15 min and then vortexed again to ensure complete extraction of the loaded photosensitizer. The nanoclusters were then magnetically separated using an external magnet, and samples of the resulting supernatant were transferred to a 96-well microplate. Absorbance measurements were recorded using a microplate reader at 405 nm, corresponding to the characteristic Soret absorption band of PPIX. The amount of loaded PPIX was measured using a calibration curve generated from standard PPIX solutions. The encapsulation efficiency (EE%) was calculated according to the following equation:

Encapsulation Efficiency (%) = (Loaded PPIX/Total PPIX added) × 100(1)

### 2.4. Physicochemical Characterization

Nanocluster morphology was investigated using transmission electron microscopy (TEM, Jeol 2100F 200 kV HRTEM, Tokyo, Japan). The hydrodynamic particle size and polydispersity index (PDI) were determined by dynamic light scattering (DLS) using a Zetasizer Nano ZS (Malvern ZS90, Worcestershire, UK) and surface charge was measured by zeta potential analysis (Malvern ZS90, Worcestershire, UK). Measurements were performed at 25 °C with the nanoparticles dispersed in distilled water.

Chemical interactions between nanocluster components and PPIX were investigated using Fourier transform infrared spectroscopy (FTIR, Bruker Optics GmbH, Ettlingen, Germany). The synthesis and detailed physicochemical characterization of the main superparamagnetic iron oxide nanoclusters (SPIONCs) were reported in our previous work [34]. Therefore, the present study primarily focuses on the characterization of mesoporous silica-coated magnetic nanoclusters (MNCs) and PPIX-loaded formulation (PPIX@MNCs) that constitute the final nanoplatform used for biological research. All analyses were performed according to established nanocluster characterization methodologies.

### 2.5. Cold Atmospheric Plasma (CAP) Treatment

The cold atmospheric plasma (CAP) source used in this study was developed by PACEM (Türkiye). The system operates with argon working gas at a temperature range of 25–40 °C, a discharge power of 360 W, and an operating frequency between 1 Hz and 10 kHz. Powered by a 32 V DC input and a 100 kV AC output, the system produces an electromagnetic power density of 20 mW/m^2^ along the x–y axis and 12 mW/m^2^ along the z-axis. The plasma source operates at approximately 1 MHz radio frequency and a voltage amplitude ranging from 0 to 50 kV. The argon flow rate was kept constant at 1 L/min, and the plasma was generated at the pin electrode tip and dispersed into the surrounding ambient air. During the process, the distance between the plasma jet nozzle and the surface of the 96-well plate was kept constant at 15 mm. The cells were exposed to CAP for 30 s under the same operating conditions. Plasma processing parameters and device configuration were kept constant throughout the study, in accordance with the protocol determined in our previous study [34].

### 2.6. Photodynamic Treatment

Following nanocluster incubation, cells treated with PPIX or PPIX@MNC were subjected to light irradiation. Photodynamic activation was performed using a visible LED light source (CATA LED, Zhongshan, China) emitting illumination in the daylight spectrum with an average luminous flux of 1500 lm and a color temperature of 3200 K. Irradiation was measured using an Nova II power meter (Ophir Optronics Ltd., Jerusalem, Israel). Cells were exposed to white light for 10 min under standardized conditions, corresponding to a total energy dose of 2.16 J/cm^2^. Irradiation parameters were selected based on previously reported PPIX-mediated photodynamic therapy protocols and kept constant throughout the experiments [37].

### 2.7. Cell Viability Assay

Human immortalized keratinocyte (HaCaT) cells were cultured in Dulbecco’s Modified Eagle Medium (DMEM; Gibco, Thermo Fisher Scientific, Grand Island, NY, USA) supplemented with 10% fetal bovine serum (FBS) and 1% penicillin-streptomycin and maintained at 37 °C in a humidified atmosphere containing 5% CO_2_.

For biological experiments, HaCaT cells were seeded into 96-well plates at a cell density of 1 × 104 cells per well and allowed to adhere for 24 h. Following cell adhesion, cultures were treated with PPIX (10, 20, 40, and 60 nM), MNC (5, 10, 20, and 30 μg mL^−1^), or PPIX@MNC formulations. Immediately after treatment, culture plates were placed on a MagnetoFACTOR-96 NdFeB magnetic plate (Chemicell, Berlin, Germany; maximum surface magnetic field strength: 0.3 T) for 15 min to create a vertical magnetic field gradient and facilitate magnetic targeting.

The cells were then assigned to one of four treatment conditions: dark, light, CAP, or combined CAP + light condition. For dark conditions, treated cells were incubated without further exposure. For photodynamic therapy, cells were incubated for 1 h after nanocluster application and magnetic targeting, followed by 10 min of white light irradiation. For CAP treatment, cells were incubated for 1 h and then exposed to CAP for 30 s without light irradiation. For combined CAP-assisted photodynamic therapy, cells were first exposed to white light for 10 min and immediately followed by a 30 s CAP treatment. After treatment, all groups were incubated for 24 or 48 h prior to analysis.

Cell viability was assessed using the Cell Count Kit-8 (CCK-8) assay. At the end of the incubation period, the culture medium was removed, and the cells were gently washed twice with phosphate-buffered saline (PBS) to remove any remaining nanoclusters and unbound compounds. Then, CCK-8 reagent was added to each well at a final concentration of 10% (*v*/*v*), and the plates were incubated at 37 °C for 2 h. Absorbance was measured at 450 nm using an Epoch microplate reader (BioTek Instruments, Inc., Winooski, Vermont, USA), and cell viability was expressed as a percentage relative to untreated control cells. Phototoxicity Index (PI) was calculated as: PI = IC50 (Dark-PPIX)/IC50 (Light-PPIX) using viability values obtained at the highest tested concentration. Similar enhancement analyses have been previously employed for evaluating PDT efficacy and nanocluster-assisted therapeutic performance. The phototoxicity index (PI) is commonly used to evaluate the selectivity and efficacy of photosensitizers following light activation. While universally accepted threshold values have not been established, PI values are generally interpreted as follows: PI < 1 indicates the absence of phototoxicity; PI values between 1 and 2 indicate weak phototoxicity; values between 2 and 5 indicate moderate phototoxicity; values between 5 and 10 indicate strong phototoxicity; and PI values greater than 10 reflect very strong phototoxicity and high photoselector status. In general, photosensitizers with PI values above 100 are considered highly effective candidates for photodynamic therapy applications [38,39].

### 2.8. ROS Determination

Extracellular ROS generation was evaluated in a cell-free culture medium under identical treatment conditions used for the biological experiments. Complete DMEM containing 10% fetal bovine serum and 1% penicillin–streptomycin was incubated with MNC, free PPIX, or PPIX@MNC at the highest tested concentrations (30 μg mL^−1^ MNC and 60 nM PPIX) for 2 h and subsequently exposed to light, CAP, or CAP-assisted photodynamic treatment. ROS levels were determined using the DCFH-DA fluorescence assay. Fluorescence intensity was measured using a GloMax microplate reader (Promega, Germany) (Ex: 460 nm, Em: 500–550 nm). ROS levels were normalized to the untreated control group.

### 2.9. Apoptosis Analysis

Apoptotic cell death was assessed using the Annexin V-FITC/Propidium Iodide (PI) Apoptotic Detection Kit (Elabscience Biotechnology Co., Ltd., Wuhan, China) according to the manufacturer’s instructions. For apoptotic assays, HaCaT cells were treated with the highest tested concentrations of MNC (30 μg mL−1), PPIX (60 nM), or PPIX@MNC under dark, light, CAP (30 s), and combined CAP + light treatment conditions. After 48 h of incubation, cells were collected, washed twice with ice-cold phosphate-buffered saline (PBS), and centrifuged at 2000× *g* for 5 min. Cell pellets were gently resuspended in 500 μL of binding buffer, followed by the addition of 5 μL of Annexin V-FITC and 5 μL of PI solution. Samples were gently mixed and incubated in the dark at room temperature for 15 min. The measurements were performed on pooled samples collected from multiple culture wells for each experimental group and analyzed as a single acquisition.

Stained cells were analyzed using a NovoCyte 3 flow cytometer (Agilent Technologies, Santa Clara, CA, USA). Fluorescence signals were detected using the FITC channel for Annexin V-FITC and the PE channel for PI. Data acquisition and analysis were performed using NovoExpress software (version 1.5.0). Cell populations were classified as viable (Annexin V−/PI−), early apoptotic (Annexin V+/PI−), late apoptotic (Annexin V+/PI+), or necrotic (Annexin V−/PI+). Representative Annexin V-FITC/PI flow cytometry dot plots obtained from a representative experiment are presented in the Supplementary Information Section to illustrate the distribution of viable, early apoptotic, late apoptotic, and necrotic cell populations following different treatments.

### 2.10. Mitochondrial Membrane Potential Analysis

Mitochondrial membrane potential (ΔΨm) was assessed using the JC-1 assay. HaCaT cells treated with MNC (30 μg mL^−1^), PPIX (60 nM), or PPIX@MNC under dark, light, CAP (30 s), and CAP-assisted photodynamic conditions were collected after 48 h of incubation. The measurements were performed on pooled samples collected from multiple culture wells for each experimental group and analyzed as a single acquisition. Cells were stained with JC-1 dye for 30 min at 37 °C in the dark according to the manufacturer’s instructions and then analyzed using a NovoCyte 3 flow cytometer (Agilent Technologies, USA). JC-1 monomers (green fluorescence) and aggregates (red fluorescence) were detected to assess changes in mitochondrial membrane potential. Prior to fluorescence analysis, cellular debris and doublets were excluded by sequential forward and side scatter (FSC/SSC) gating followed by singlet gating. The same gating strategy was applied to all samples. Flow cytometry data were analyzed using NovoExpress software (version 1.5.0). Representative flow cytometry plots are provided in the Supplementary Information [40,41].

### 2.11. Statistical Analysis

All experiments were performed in quadruplicate (*n* = 4) and expressed as mean ± standard deviation (SD). Statistical analyses were performed using GraphPad Prism 8 software. Comparisons among multiple groups were performed using the Kruskal–Wallis test followed by Dunn’s multiple comparisons test. A *p* value < 0.05 was considered statistically significant.

## 3. Results

### 3.1. Physicochemical Characterization of MNC and PPIX@MNC

**Figure 2:**
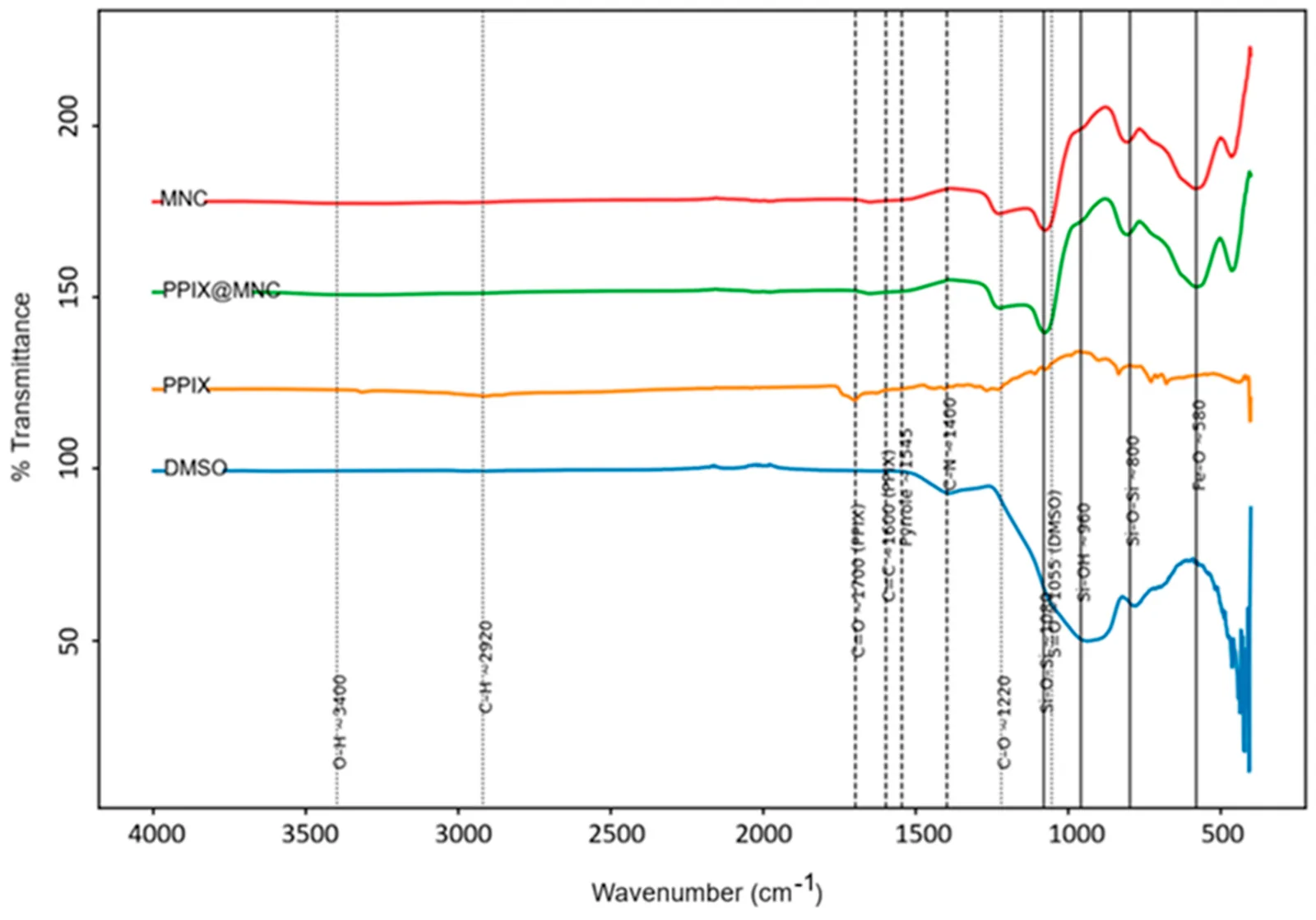
FTIR spectra of DMSO, PPIX, MNC and PPIX-loaded MNC (MNC–PPIX). See Table 1 for sample details.

FTIR analyses confirmed successful loading of PPIX onto mesoporous silica-coated iron oxide nanoclusters in Figure 2. Characteristic Si–O–Si asymmetric and symmetric stretching vibrations were observed around 1080 cm^−1^ and 800 cm^−1^, respectively. The Si–OH band around 960 cm^−1^ and the Fe–O vibration band near 580 cm^−1^ confirmed the presence of silica and iron oxide structures.

PPIX-associated characteristic peaks including the carbonyl stretching band around 1700 cm^−1^, aromatic C=C vibrations around 1600 cm^−1^, pyrrole-related bands near 1545 cm^−1^, and C–N vibrations around 1400 cm^−1^ were also identified in PPIX@MNC samples, confirming incorporation of PPIX. After the loading process, the nanoclusters were thoroughly washed with ethanol and distilled water to remove residual DMSO before FTIR measurements. Therefore, the absorption band in the 1000–1100 cm^−1^ region was mainly attributed to the Si–O–Si asymmetric stretching vibration of the mesoporous silica skeleton. Although a negligible contribution from residual DMSO cannot be completely excluded, it is unlikely to have materially influenced the observed biological responses.

**Figure 3:**
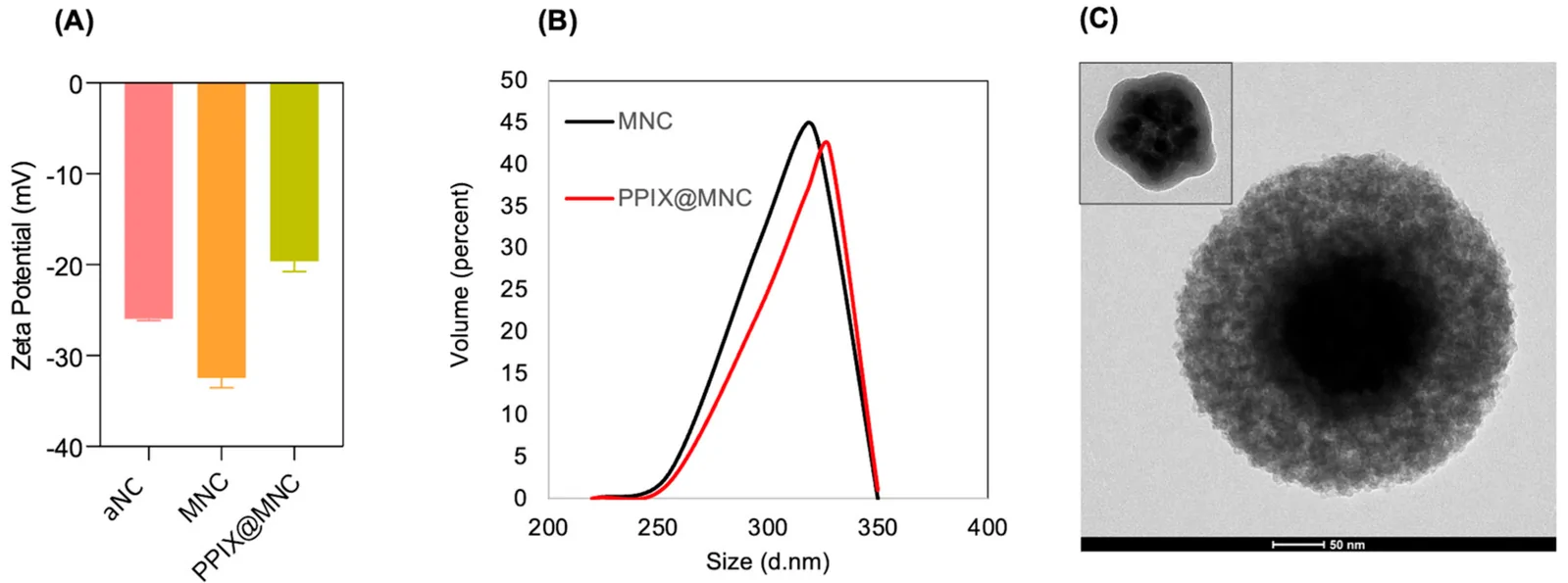
(**A**) Zeta potential of aNC, MNC and PPIX@MNC, (**B**) DLS analysis of MNC and PPIX@MNC, (**C**) TEM micrograph of MNC. Inset in (**C**) shows TEM micrographs of aNC. See Table 1 for sample details.

Zeta potential analysis showed that amorphous silica nanoclusters (aNC) exhibited a zeta potential of −25.9 ± 0.2 mV. Mesoporous silica-coated magnetic nanoclusters (MNC) exhibited a surface charge of −32.4 ± 1.0 mV, while PPIX loading shifted the zeta potential to −19.6 ± 1.1 mV, as shown in Figure 3A.

DLS analyses demonstrated hydrodynamic diameters of 314.9 ± 50.1 nm (PDI = 0.60) for MNC and 361.5 ± 51.3 nm (PDI = 0.50) for PPIX-loaded nanoclusters (PPIX@MNC) (Figure 3B). Quantitative analysis of TEM images revealed that the average diameter of the Fe_3_O_4_ nanoclusters (NC) core was 155 ± 14 nm. Following the deposition of the compact amorphous silica shell (aNC), the average particle diameter increased to 211 ± 11 nm (Figure 3C, inset), while the average diameter of the final MNC was 310 ± 24 nm in Figure 3C. Furthermore, the thicknesses of the amorphous silica shell and the mesoporous silica layer were determined to be 21 ± 1.4 nm and 57 ± 4.0 nm, respectively.

#### PPIX-Loaded Nanoclusters (PPIX@MNC)

The loading of PPIX onto MNCs was evaluated at MNC:PPIX mass ratios of 20:1, 50:1, and 100:1 (*w*/*w*). Among the tested formulations, the 20:1 ratio achieved the highest PPIX loading. Quantitative analysis based on the calibration curve revealed an encapsulation efficiency of 2.23% (*w*/*w*) for this formulation. The PPIX loading contents were determined to be 1.12, 0.51, and 0.39 ng per 1 μg of MNC for the 20:1, 50:1, and 100:1 formulations, respectively. Based on these results, the 20:1 formulation was selected for all subsequent experiments because it provided the highest PPIX loading among the tested formulations.

### 3.2. Cell Viability Analysis

Cell viability was assessed using the CCK-8 assay and expressed as a percentage relative to untreated control cells. IC50 values were determined by linear interpolation between the concentrations immediately above and below 50% viability.

**Figure 4:**
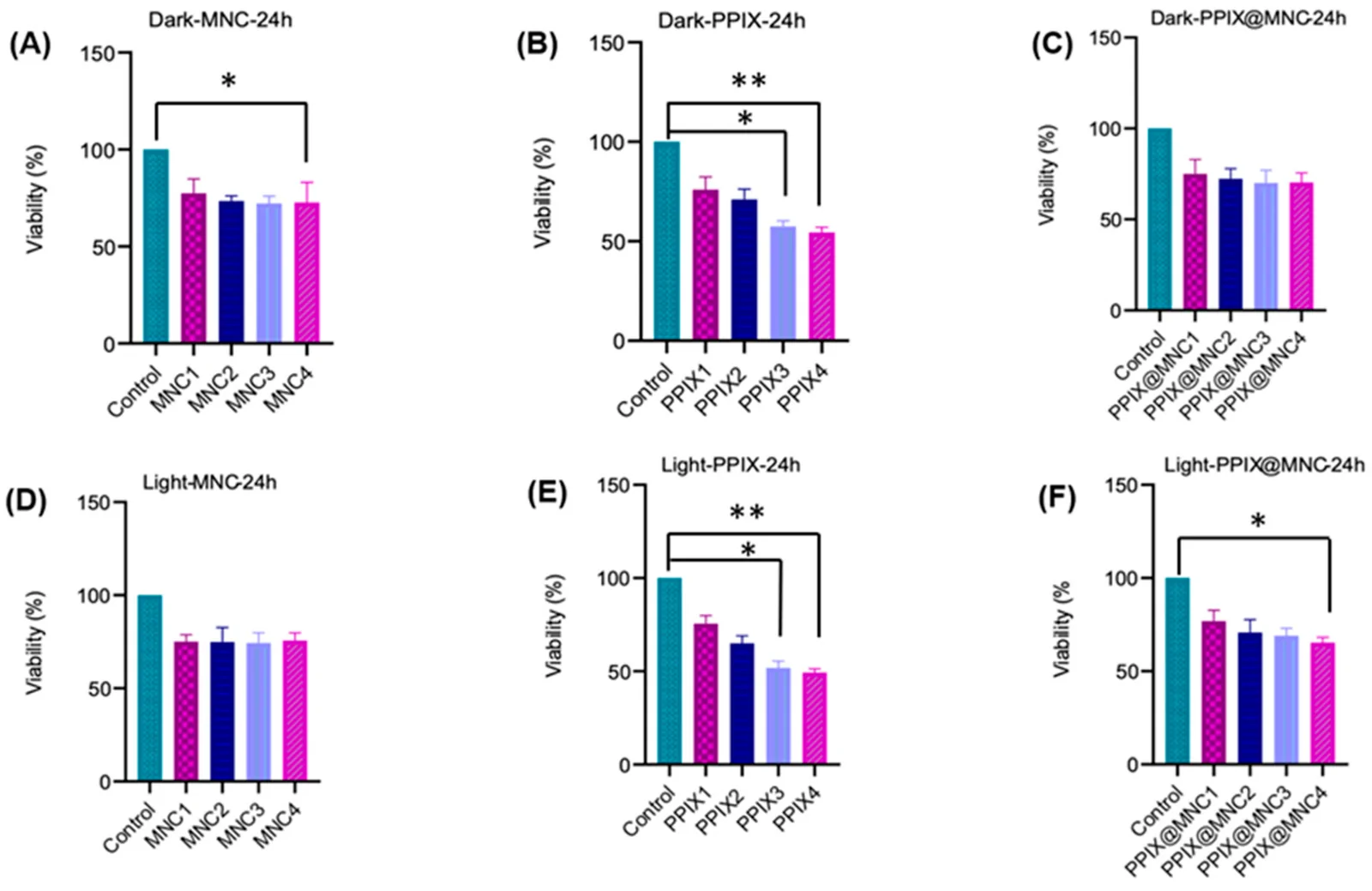
CCK-8 analysis of HaCaT cell viability after 24 h of incubation. Cells were treated with MNC (5–30 µg/mL), PPIX (10–60 nM), or PPIX@MNC under dark or light (10 min irradiation) conditions. (**A**) Dark-MNC, (**B**) Dark-PPIX, (**C**) Dark-PPIX@MNC, (**D**) Light-MNC, (**E**) Light-PPIX, and (**F**) Light-PPIX@MNC. Data are expressed as mean ± SD (*n* = 4) and presented as viability relative to untreated controls. Asterisks indicate statistically significant differences compared with the control group. Statistical significance was defined as follows: *p* < 0.05 (*), *p* < 0.005 (**). See Table 1 for sample details.

At 24 h, no IC50 values were observed for the Dark-MNC, Dark-PPIX@MNC, Light-MNC, or Light-PPIX@MNC groups within the tested concentration ranges (Figure 4). Under light irradiation, the Light-PPIX group (Figure 4E) exhibited an IC50 value of 54.7 nM, calculated by linear interpolation between 40 and 60 nM. No IC50 value was reached for the Dark-PPIX group (Figure 4B).

**Figure 5:**
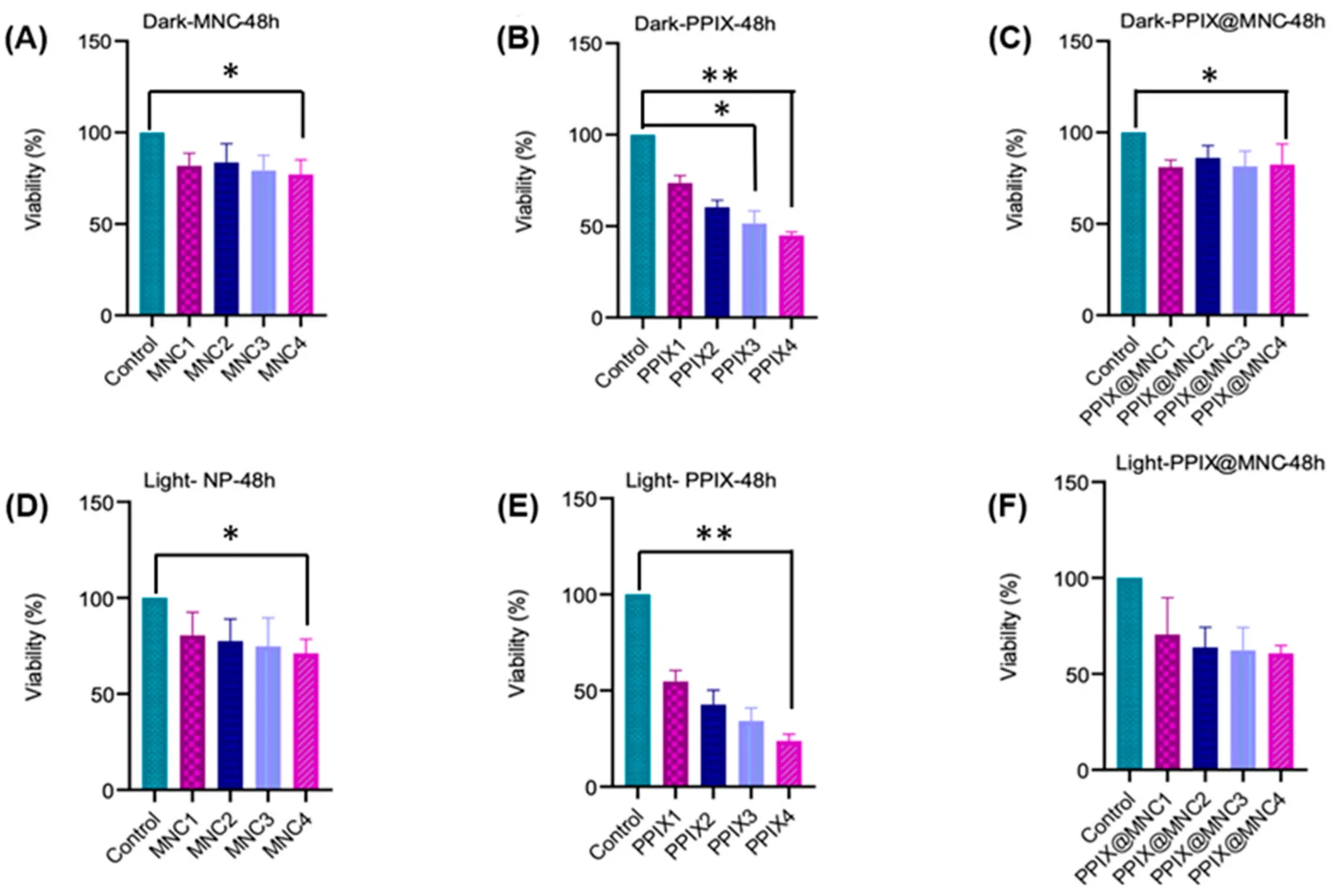
CCK-8 analysis of HaCaT cell viability after 48 h of incubation. Cells were treated with MNC (5–30 µg/mL), PPIX (10–60 nM), or PPIX@MNC under dark or light (10 min irradiation) conditions. (**A**) Dark-MNC, (**B**) Dark-PPIX, (**C**) Dark-PPIX@MNC, (**D**) Light-MNC, (**E**) Light-PPIX, and (**F**) Light-PPIX@MNC. Data are expressed as mean ± SD (*n* = 4) and presented as viability relative to untreated controls. Asterisks indicate statistically significant differences compared with the control group. Statistical significance was defined as follows: *p* < 0.05 (*), *p* < 0.005 (**). See Table 1 for sample details.

Following 48 h of incubation under dark conditions, no IC50 values were observed for the Dark-MNC (Figure 5A) or Dark-PPIX@MNC (Figure 5C) groups within the tested concentration ranges. In contrast, Dark-PPIX treatment (Figure 5B) resulted in a concentration-dependent reduction in cell viability, yielding an IC50 value of 44.4 ± 3.5 nM.

Under light irradiation at 48 h, no IC50 value was observed for the Light-MNC group (Figure 5D). The Light-PPIX group (Figure 5E) demonstrated a pronounced concentration-dependent decrease in viability, with an IC50 value of 14 ± 2 nM. No IC50 value was reached for the Light-PPIX@MNC group (Figure 5F); however, cell viability decreased to 60.7 ± 4.13% at the highest tested concentration.

**Figure 6:**
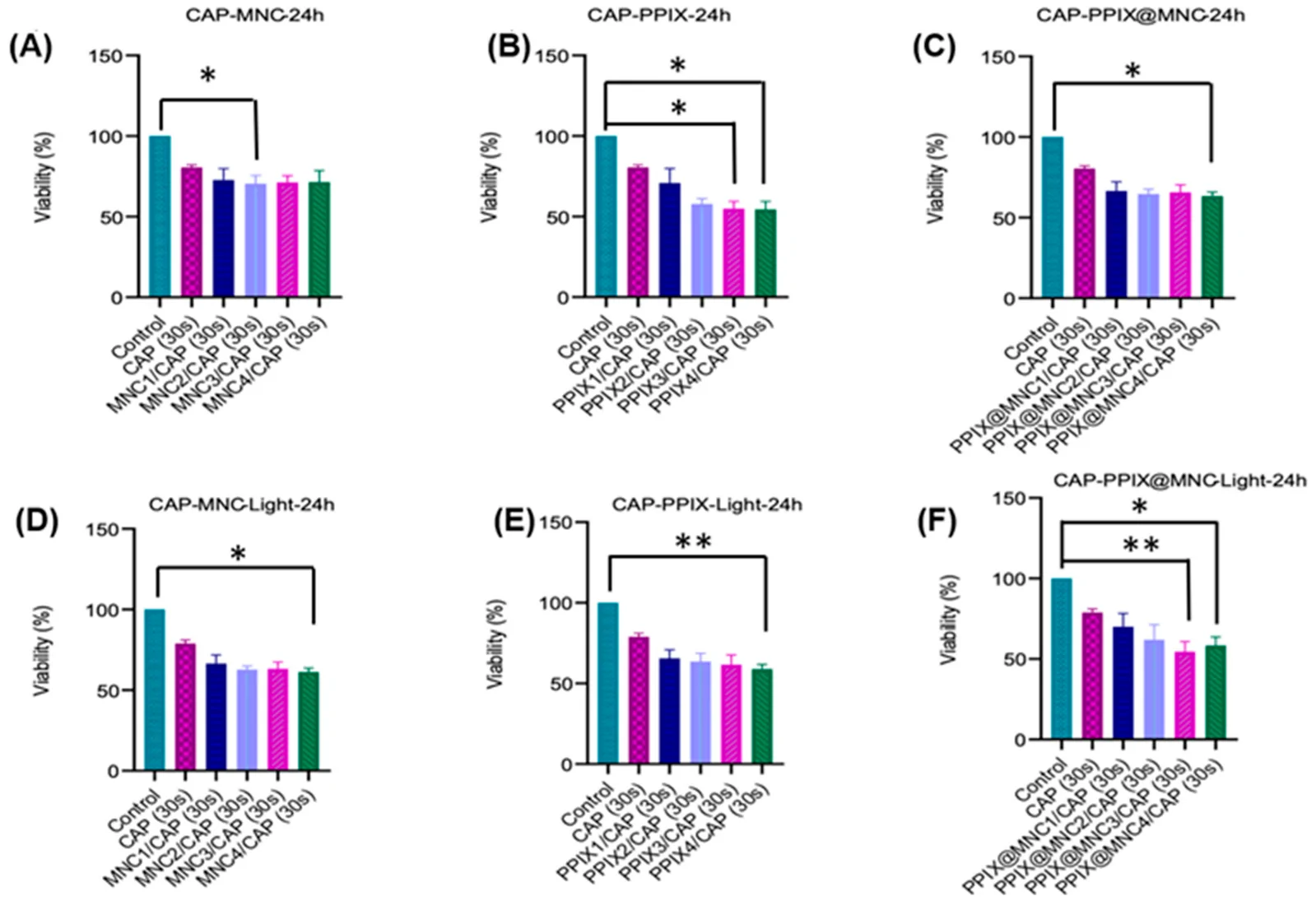
CCK-8 analysis of HaCaT cell viability after 24 h of incubation. Cells were treated with MNC (5–30 µg/mL), PPIX (10–60 nM), or PPIX@MNC in the presence of 30 s CAP, under dark or light (10 min irradiation) conditions. (**A**) CAP-MNC, (**B**) CAP-PPIX, (**C**) CAP-PPIX@MNC, (**D**) CAP-MNC-Light, (**E**) CAP-PPIX-Light, and (**F**) CAP-PPIX@MNC-Light. Data are presented as mean ± SD (*n* = 4) and expressed as percentage viability relative to untreated controls. Asterisks indicate statistically significant differences compared with the control group. Statistical significance was defined as follows: *p* < 0.05 (*), *p* < 0.005 (**). See Table 1 for sample details.

The phototoxicity index (PI), calculated as the ratio of the Dark-PPIX IC50 to the Light-PPIX IC50, was 3.17, indicating increased cytotoxicity following photoactivation. After 30 s CAP exposure, no IC50 values were observed in any CAP-treated groups following 24 h incubation. Although moderate reductions in viability were detected, cell survival remained above 50% across all tested concentrations (Figure 6A–C). Among the CAP-assisted groups, CAP-Light-PPIX (Figure 6E) exhibited an IC50 value of 46.1 ± 3.0 nM, suggesting that plasma treatment enhanced the cytotoxic activity of PPIX compared with CAP treatment alone. However, the tested concentration range did not reduce cell viability to 50% (Figure 6D); therefore, the IC50 value could not be calculated.

**Figure 7:**
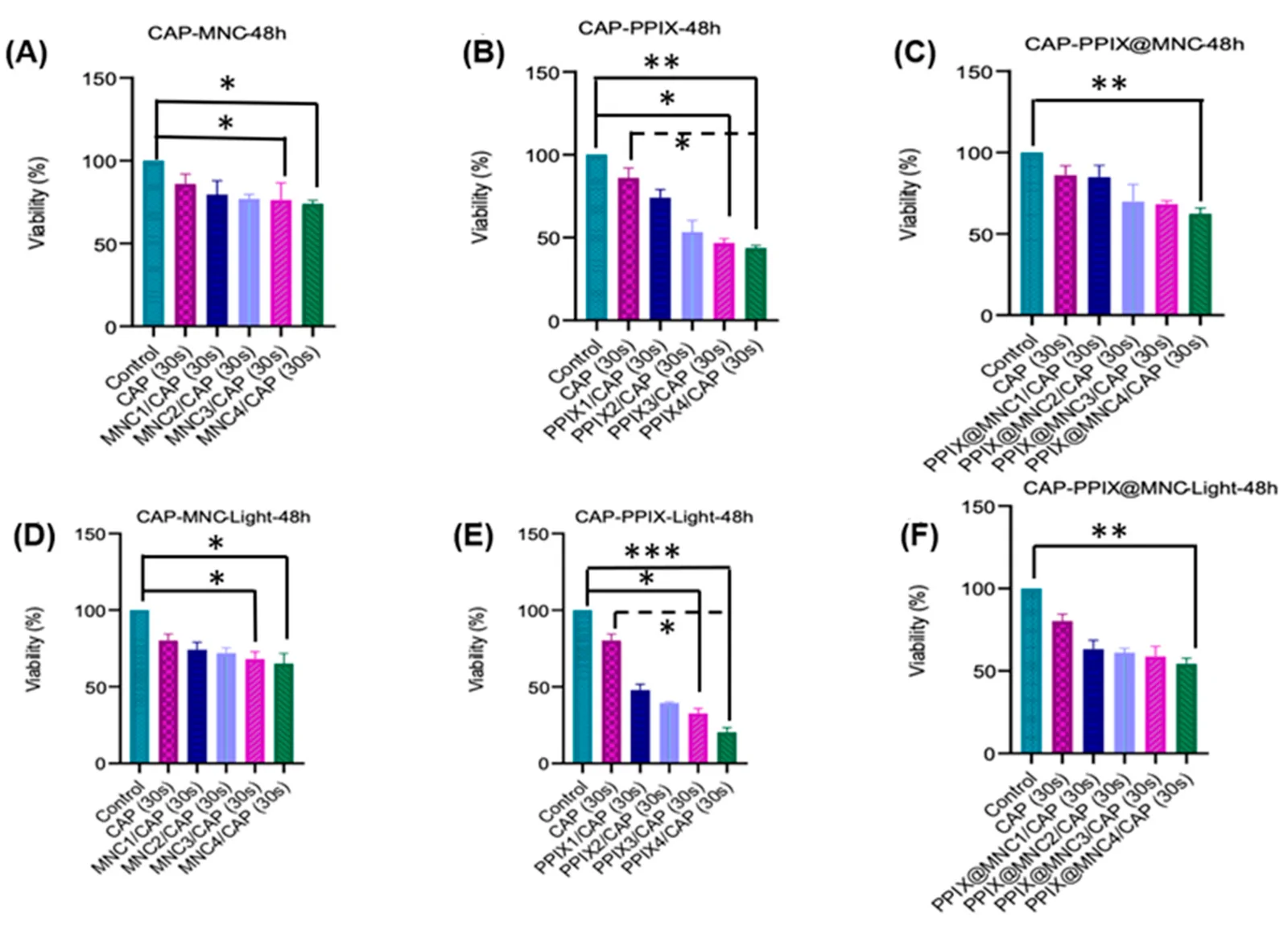
CCK-8 analysis of HaCaT cell viability after 48 h of incubation. Cells were treated with MNC (5–30 µg/mL), PPIX (10–60 nM), or PPIX@MNC in the presence of 30 s CAP, under dark or light (10 min irradiation) conditions. (**A**) CAP-MNC, (**B**) CAP-PPIX, (**C**) CAP-PPIX@MNC, (**D**) CAP-MNC-Light, (**E**) CAP-PPIX-Light, and (**F**) CAP-PPIX@MNC-Light. Data are presented as mean ± SD (*n* = 4) and expressed as percentage viability relative to untreated controls. Asterisks indicate statistically significant differences compared with the control group unless otherwise specified by connecting lines. Statistical significance was defined as follows: *p* < 0.05 (*), *p* < 0.005 (**) and *p* < 0.005 (***). See Table 1 for sample details.

After 48 h of incubation, no IC50 values were observed for the CAP-MNC (Figure 7A) or CAP-PPIX@MNC (Figure 7C) groups within the tested concentration ranges. In contrast, CAP-PPIX (Figure 7B) exhibited an IC50 value of 30.0 ± 4.5 nM, demonstrating enhanced cytotoxicity compared with Dark-PPIX.

Under combined CAP and light irradiation conditions, no IC50 value was reached for either the CAP-Light-MNC (Figure 7D) or CAP-Light-PPIX@MNC (Figure 7F) groups. Nevertheless, CAP-Light-PPIX (Figure 7E) produced the strongest cytotoxic response among all treatment groups, with an estimated IC50 value of 9.6 ± 1.1 nM.

No IC50 value was observed for the CAP-Light-PPIX@MNC formulation within the tested concentration range. At the highest concentration tested (60 nM), cell viability remained at 54.39 ± 3.37% (Figure 7F).

### 3.3. Apoptosis Assessment

Flow cytometric analysis demonstrated marked differences in apoptotic responses among the experimental groups in Figures S1 and S2 (Supplementary File). Control cells exhibited high viability (99.8%) with minimal apoptotic or necrotic populations, confirming normal cellular homeostasis (Figures S1A and S2A).

MNC-treated groups exhibited comparatively lower apoptotic activity. Light-MNC4 treatment maintained 64.4% viable cells (Figure S2B), whereas Dark-MNC4 preserved 79.5% cell viability (Figure S1B). Among the treatment groups, light-activated PPIX induced substantial apoptotic cell death, with only 22.5% viable cells and 59.7% late apoptotic cells (Figure S2C). Dark-PPIX treatment also promoted apoptosis, resulting in 48.2% late apoptosis (Figure S1C).

PPIX@MNC formulations showed reduced apoptotic responses compared with free PPIX. Under light irradiation, PPIX@MNC treatment resulted in 16.4% late apoptosis (Figure S2D), which was markedly lower than that observed for free PPIX (Figure S2C). Similar patterns were observed under both dark and CAP-treated conditions.

CAP treatment alone induced a moderate apoptotic response, characterized by 5.5% late apoptosis (Figure S2E). In contrast, the combination of CAP and PPIX markedly increased apoptotic cell death. The CAP-Light-PPIX group exhibited the highest late apoptotic population (74.3%) among all experimental groups (Figure S2G).

Overall, apoptosis analysis demonstrated that photoactivated PPIX was the most potent inducer of apoptotic cell death, whereas incorporation of PPIX into MNCs reduced the apoptotic response relative to the free photosensitizer.

### 3.4. Mitochondrial Membrane Potential Assessment

Mitochondrial membrane potential (ΔΨm) was evaluated by JC-1 staining and flow cytometric analysis. Untreated control cells predominantly exhibited a polarized mitochondrial profile, indicating preserved mitochondrial integrity. In contrast, PPIX-containing treatments induced substantial alterations in mitochondrial membrane potential distribution under dark, light, CAP, and CAP-assisted photodynamic conditions (Figure S3).

Free PPIX treatment resulted in a marked shift from the polarized mitochondrial population toward altered mitochondrial states, with more pronounced changes observed following light irradiation and CAP-assisted photodynamic treatment. The CAP-Light-PPIX group exhibited the most pronounced disruption of mitochondrial membrane potential among the free PPIX-treated groups.

MNC-treated cells largely maintained a polarized mitochondrial profile, suggesting limited effects on mitochondrial function. Similarly, PPIX@MNC formulations generally exhibited less pronounced mitochondrial alterations than free PPIX under comparable treatment conditions. However, CAP-Light-PPIX@MNC treatment produced noticeable changes in mitochondrial membrane potential distribution compared with the corresponding dark and light-treated nanocarrier groups.

Overall, JC-1 analysis demonstrated that PPIX-containing treatments, particularly under photodynamic and CAP-assisted photodynamic conditions, induced significant mitochondrial membrane potential alterations relative to untreated controls, whereas MNC alone had comparatively minor effects on mitochondrial polarization.

### 3.5. ROS Generation

**Figure 8:**
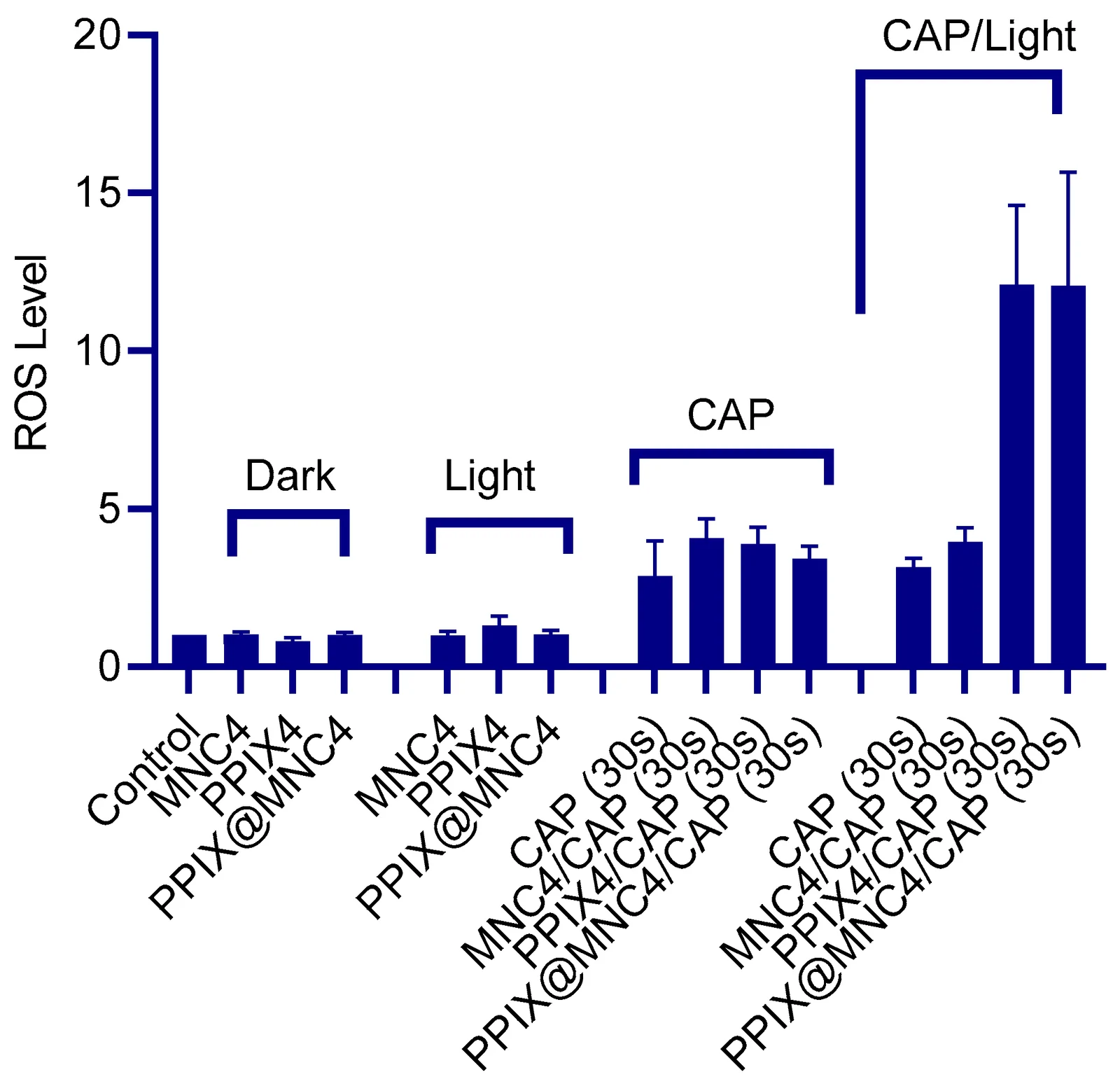
Normalized ROS levels measured in the culture medium of HaCaT cells treated with MNC4, PPIX4, and PPIX@MNC4 under different experimental conditions: dark, light irradiation, CAP, combined CAP and light irradiation. ROS levels were normalized relative to the untreated control group. Data are presented as mean ± SD (*n* = 4). See Table 1 for sample details.

ROS production was assessed using the DCFH-DA assay, and results are presented in Figure 8. Under dark conditions, MNC and PPIX@MNC produced ROS levels similar to the untreated control group. Light irradiation increased ROS production in the PPIX-containing groups, while CAP treatment further increased ROS levels. The highest ROS signal was observed in the CAP-Light-PPIX group. No statistically significant difference was found between the CAP-Light-PPIX and CAP-Light-PPIX@MNC groups; however, both groups exhibited significantly higher ROS levels compared to the other treatment groups (*p* < 0.05).

## 4. Discussion

The development of multifunctional nanoplatforms capable of enhancing photodynamic efficacy while minimizing undesirable side effects remains a major objective in nanomedicine. In the present study, mesoporous silica-coated iron oxide nanoclusters were successfully loaded with PPIX and subsequently evaluated under dark, light, CAP, and CAP-assisted photodynamic conditions. The obtained results demonstrate that free PPIX remained the most potent cytotoxic and pro-apoptotic formulation, whereas incorporation into the MNC carrier reduced acute biological activity while preserving responsiveness to external stimulation.

FTIR analysis confirmed the successful incorporation of PPIX into the mesoporous silica-coated magnetic nanoclusters through the simultaneous presence of characteristic porphyrin and silica/iron oxide absorption bands (Figure 2). The hydrodynamic diameter determined by DLS (Figure 3B) was slightly larger than the average particle size measured by TEM (Figure 3C). This difference was expected because DLS measures the hydrodynamic diameter of nanoparticles dispersed in a liquid medium, while TEM determines the physical diameter of dried particles [42]. Furthermore, another study highlighted that the large differences between DLS and TEM are not an inherent characteristic of these techniques, and that both methods can provide very similar particle size values when measurements are performed and interpreted correctly [43]. The zeta potential shifted from −32.4 ± 1.0 mV to −19.6 ± 1.1 mV (Figure 3A). The increase in particle size together with the reduction in the magnitude of the negative zeta potential indicates successful loading of PPIX onto the mesoporous silica surface. Although PPIX possesses negatively charged carboxylate groups under aqueous conditions, adsorption through hydrogen bonding, hydrophobic interactions, and possible coordination with surface sites can partially shield the original silica surface charge. In addition, formation of an organic PPIX layer shifts the electrokinetic slipping plane away from the silica surface, resulting in a less negative measured zeta potential. Furthermore, partial aggregation of surface-bound PPIX molecules within or around the mesoporous structure may also contribute to the observed decrease in the absolute zeta potential [44,45]. Similar alterations in physicochemical properties, including changes in surface charge following guest molecule incorporation, have been widely reported for mesoporous silica-based delivery systems and are commonly used as indicators of successful loading and host–guest interactions within the porous framework [46,47]. Furthermore, the hydrodynamic diameters obtained by DLS remained within the nanoscale range after loading, indicating that PPIX incorporation did not substantially alter particle size or induce extensive aggregation. Preservation of nanoscale dimensions and colloidal stability following cargo loading has previously been described as a favorable characteristic of mesoporous silica nanocarriers for biomedical applications [48,49].

Although the encapsulation efficiency of PPIX was 2.23% (*w*/*w*), this value should be interpreted considering the physicochemical characteristics of PPIX and the loading mechanism employed. PPIX is a highly hydrophobic photosensitizer with limited aqueous solubility and a strong tendency to undergo π–π stacking and aggregation, which can restrict its adsorption into mesoporous silica pores. Consequently, adsorption-based loading strategies frequently result in relatively modest loading efficiencies compared with covalent conjugation or in situ encapsulation approaches. Nevertheless, for photodynamic therapy, therapeutic efficacy depends predominantly on efficient intracellular delivery, subcellular localization, and photoactivation of the photosensitizer rather than solely on the absolute loading amount. In the present study, despite the relatively low loading efficiency, PPIX@MNC exhibited measurable photodynamic activity, particularly under CAP-assisted conditions, indicating that the achieved loading was sufficient to produce biologically meaningful responses. Future optimization of pore architecture, surface chemistry, and loading strategies may further improve loading efficiency while preserving the favorable physicochemical properties and stability of the nanoplatform.

The cell viability results demonstrated a clear dependence on treatment modality. MNC alone exhibited limited cytotoxicity under all experimental conditions, supporting previous reports describing mesoporous silica-coated iron oxide nanomaterials as relatively biocompatible systems at comparable concentrations [50,51]. In contrast, free PPIX displayed concentration-dependent cytotoxicity, particularly after light activation. The reduction in the IC50 value from 44.4 ± 3.5 nM under dark conditions (Figure 5B) to 14 ± 2 nM following light irradiation (Figure 5E) yielded a phototoxicity index of 3.2, confirming effective photoactivation of PPIX. This observation is consistent with the established mechanism of porphyrin-mediated photodynamic therapy, in which excitation of PPIX results in efficient singlet oxygen and ROS generation, ultimately triggering oxidative cellular damage [16].

Interestingly, PPIX@MNC formulations did not reach IC50 values within the investigated concentration range, even under light irradiation (Figure 4). The reduced cytotoxicity and apoptotic activity observed for PPIX@MNC compared with free PPIX suggest that incorporation into the mesoporous carrier altered the immediate biological availability of the photosensitizer. Similar behavior has been reported for silica-based photosensitizer delivery systems, where encapsulation can decrease acute toxicity while enabling more controlled release and improved stability of the loaded cargo [22]. The lower apoptotic fractions observed in the PPIX@MNC groups support this interpretation.

CAP exposure further influenced treatment efficacy. Although CAP alone produced only moderate biological effects, CAP-assisted treatments consistently demonstrated enhanced activity relative to corresponding non-CAP groups (Figure 6). After 48 h incubation, CAP-PPIX (Figure 7B) exhibited a lower IC50 value than Dark-PPIX (Figure 5B), indicating that plasma-generated reactive species contributed to the cytotoxic response. The strongest effect was observed in the CAP-Light-PPIX group (Figure 7E), which yielded an IC50 value of 9.6 ± 1.1 nM.

Augmented biological response observed in the CAP-supported PDT group. The lower IC50 value obtained for CAP-Light-PPIX compared with both CAP-PPIX and Light-PPIX alone indicates that simultaneous exposure to CAP-generated reactive species and photoactivated PPIX produced a greater biological effect than either treatment modality individually. Previous studies have demonstrated that CAP generates a complex mixture of reactive oxygen and nitrogen species capable of enhancing intracellular oxidative stress and sensitizing cells to ROS-mediated therapies [52,53]. The present findings are consistent with these reports and indicate that CAP can potentiate the biological activity of photoactivated PPIX.

The elevated ROS levels, increased apoptotic populations, and pronounced mitochondrial membrane potential alterations observed in the CAP-Light-PPIX group further support the existence of a potentiated interaction between plasma treatment and photodynamic activation (Figure 8). The ROS measurements further support this interpretation. While MNC and PPIX@MNC generated ROS levels comparable to untreated controls under dark conditions, photoactivation markedly increased ROS production in PPIX-containing groups. The highest ROS accumulation was observed following CAP-assisted photodynamic treatment. Importantly, both CAP-Light-PPIX and CAP-Light-PPIX@MNC exhibited significantly elevated ROS levels compared with all other groups, highlighting the critical role of oxidative stress in mediating treatment responses. Since ROS generation represents the primary mechanism underlying both CAP-induced biological effects and photodynamic therapy, the observed increase is likely responsible for the enhanced cytotoxicity and apoptosis detected in these groups [54].

Apoptosis analysis revealed a strong correlation between ROS production and cell death. Free PPIX induced substantial late apoptosis following photoactivation, whereas CAP-Light-PPIX produced the highest apoptotic response among all experimental groups (Figures S1 and S2). In contrast, MNC-treated cells maintained relatively high viability and low apoptotic fractions. These findings suggest that oxidative stress generated by photoactivated PPIX represents a major driver of apoptotic cell death and that CAP further amplifies this response. Similar increases in late apoptotic populations following combined ROS-generating therapies have been reported in both plasma medicine and photodynamic therapy studies [53,55].

Changes in mitochondrial membrane potential provided additional evidence of oxidative stress-mediated cellular responses (Figure S3). JC-1 analysis demonstrated substantial alterations in mitochondrial polarization in PPIX-treated cells, particularly under light and CAP-assisted photodynamic conditions (Figure S3B). In contrast, MNC-treated cells largely preserved mitochondrial polarization. Mitochondria are highly susceptible to ROS-induced damage, and disruption of mitochondrial membrane potential is considered an early hallmark of apoptosis initiation [55]. Therefore, the mitochondrial alterations observed in the present study are consistent with the elevated ROS levels and increased apoptotic responses detected in PPIX-containing groups.

The Fe_3_O_4_ core was incorporated not only as a structural support for the mesoporous silica shell but also to enable magnetic targeting. Superparamagnetic iron oxide nanoparticles are well recognized for their ability to respond efficiently to an external magnetic field while exhibiting negligible remanent magnetization after removal of the field, thereby allowing magnetic guidance without permanent aggregation. Consequently, the application of an external magnetic field can increase the local concentration of nanoparticles at the cell surface, enhancing nanoparticle–cell interactions and promoting cellular internalization [56]. In the present study, an external permanent magnet was applied for 15 min prior to treatment, which likely increased the local accumulation of PPIX@MNCs around HaCaT cells and facilitated their uptake before CAP-assisted photodynamic activation. In addition to magnetic guidance, the physicochemical characteristics of nanoparticles—including their size, shape, and surface properties—play a pivotal role in determining cellular uptake efficiency, intracellular trafficking, and biological responses. Rather than being governed by a single optimal size, nanoparticle internalization depends on the interplay between particle dimensions, morphology, surface chemistry, and the specific endocytic pathways of the target cells [57]. Therefore, besides magnetic guidance, the physicochemical characteristics of the mesoporous silica-coated iron oxide nanoclusters employed in this study likely contributed to enhanced nanoparticle–cell interactions and may have facilitated intracellular delivery of PPIX, thereby contributing to the observed biological responses following CAP-assisted photodynamic treatment.

Taken together, the present findings indicate that free PPIX remains highly responsive to both photodynamic activation and CAP treatment, resulting in enhanced ROS generation, mitochondrial dysfunction, and apoptotic cell death. Although loading into mesoporous silica-coated magnetic nanoclusters reduced the immediate cytotoxicity of PPIX, the nanocarrier system preserved responsiveness to external stimuli while potentially providing improved stability and controlled photosensitizer delivery. These characteristics may be advantageous for future optimization of CAP-assisted photodynamic nanomedicine platforms.

## 5. Conclusions

In conclusion, mesoporous silica-coated iron oxide nanoclusters were successfully synthesized and employed as carriers for protoporphyrin IX, yielding a multifunctional PPIX@MNC nanoplatform with preserved nanoscale characteristics and favorable physicochemical properties. PPIX loading was confirmed by FTIR, zeta potential, and DLS analyses, demonstrating successful incorporation of the photosensitizer into the mesoporous structure.

Biological evaluation revealed that free PPIX exhibited the highest photodynamic activity, characterized by increased ROS generation, mitochondrial membrane depolarization, apoptosis induction, and reduced cell viability following light irradiation. CAP treatment further enhanced these responses, and the combination of CAP and photodynamic activation produced the strongest biological effect, resulting in the lowest IC50 value, the highest ROS accumulation, and the greatest apoptotic response among all treatment groups. The calculated combination index values confirmed an enhanced interaction between CAP-generated reactive species and PPIX-mediated photodynamic activity.

Although encapsulation of PPIX within mesoporous silica-coated magnetic nanoclusters reduced acute cytotoxicity and apoptotic responses compared with free PPIX, the nanocarrier maintained sensitivity to both light irradiation and CAP exposure. These findings suggest that the mesoporous carrier modulates the immediate biological availability of PPIX while potentially providing improved stability and more controlled photosensitizer delivery.

As this study was conducted using non-tumorigenic HaCaT keratinocytes, the present findings should be regarded as an initial in vitro feasibility assessment rather than evidence of anticancer efficacy or tumor selectivity. Future studies investigating photosensitizer release behavior, cellular uptake mechanisms, and therapeutic efficacy in appropriate cancer cell models, together with evaluations of tumor selectivity, will provide a more comprehensive assessment of the translational potential of this nanoplatform.

## Figures and Tables

**Table 1:** List of abbreviations used for naming various generations of samples.

Abbreviation	Description
aNC	Amorphous silica-coated iron oxide nanoclusters
MNC	Mesoporous silica-coated iron oxide nanoclusters
PPIX	Protoporphyrin IX
CAP	Cold Atmospheric Plasma
MNC1	5 µg/mL
MNC2	10 µg/mL
MNC3	20 µg/mL
MNC4	30 µg/mL
PPIX1	10 nM
PPIX2	20 nM
PPIX3	40 nM
PPIX4	60 nM
MNC1/CAP	CAP and 5 µg/mL MNC combination, 30 s
MNC2/CAP	CAP and 10 µg/mL MNC combination, 30 s
MNC3/CAP	CAP and 20 µg/mL MNC combination, 30 s
MNC4/CAP	CAP and 30 µg/mL MNC combination, 30 s
PPIX1/CAP	CAP and 10 nM PPIX combination, 30 s
PPIX2/CAP	CAP and 20 nM PPIX combination, 30 s
PPIX3/CAP	CAP and 40 nM PPIX combination, 30 s
PPIX4/CAP	CAP and 60 nM PPIX combination, 30 s
PPIX@MNC1/CAP	CAP, 5 µg/mL MNC, and 10 nM PPIX combination, 30 s
PPIX@MNC2/CAP	CAP, 10 µg/mL MNC, and 20 nM PPIX combination, 30 s
PPIX@MNC3/CAP	CAP, 20 µg/mL MNC, and 40 nM PPIX combination, 30 s
PPIX@MNC4/CAP	CAP, 30 µg/mL MNC, and 60 nM PPIX combination, 30 s

## Data Availability

All data generated or analyzed during this study are included in this published article and its Supplementary Information (SI).

## Supplementary Materials

The following supporting information can be downloaded at: https://www.mdpi.com/article/10.3390/nano16161012/s1, Figure S1: Representative Annexin V-FITC/PI flow cytometry plots of HaCaT cells under dark conditions: (A) Control, (B) MNC4, (C) PPIX4, (D) PPIX4@MNC4, (E) CAP, (F) CAP-MNC4, (G) CAP-PPIX4, and (H) CAP-PPIX4@MNC4. Cell populations were classified as viable, early apoptotic, late apoptotic, or necrotic based on Annexin V-FITC and propidium iodide staining after exclusion of debris and doublets using FSC/SSC and singlet gating; Figure S2: Representative Annexin V-FITC/PI flow cytometry plots of HaCaT cells under light conditions: (A) Control, (B) MNC4, (C) PPIX4, (D) PPIX4@MNC4, (E) CAP, (F) CAP-MNC4, (G) CAP-PPIX4, and (H) CAP-PPIX4@MNC4. Cell populations were classified as viable, early apoptotic, late apoptotic, or necrotic based on Annexin V-FITC and propidium iodide staining after exclusion of debris and doublets using FSC/SSC and singlet gating; Figure S3: Population-based JC-1 red/green mitochondrial membrane potential ratio was calculated as the ratio of polarized mitochondria (Q2-2) to depolarized mitochondria (Q2-4). (A) Dark-treated groups. (B) Light-treated groups.
