# Supplementary material for: Cold Atmospheric Plasma Potentiates the Photodynamic Effects of Protoporphyrin IX-Loaded Mesoporous Silica-Coated Iron Oxide Nanoclusters in HaCaT Cells
Source: Nanomaterials (Basel). 2026 Aug 17;16(16):1012. doi: 10.3390/nano16161012 (PMC13515948; doi:10.3390/nano16161012)
Supplement: Supplementary file 1 [file nanomaterials-16-01012-s001.zip › nanomaterials-4463934-supplementary.pdf]

Supplementary Information

Cold Atmospheric Plasma Potentiates the Photodynamic Effects of Protoporphyrin IX-Loaded Mesoporous Silica-Coated Iron Oxide Nanoclusters in HaCaT Cells

Demet Erdag [1,2], Harun Basoglu [3,*], Leman Yalcintepe [4,*] and Muhammet S. Toprak [2,*]

1. Department of Computer Programming, Vocational School, Biruni University, 34015 Istanbul, Türkiye
2. Department of Applied Physics, KTH Royal Institute of Technology, SE-106 91 Stockholm, Sweden; derdag@kth.se
3. Department of Biophysics, Faculty of Medicine, Bilecik Seyh Edebali University, 11100 Bilecik, Türkiye
4. Department of Biophysics, Faculty of Medicine, Istanbul University, 34093 Istanbul, Türkiye

* Correspondence: harun.basoglu@bilecik.edu.tr (H.B.); lemany@istanbul.edu.tr (L.Y.); toprak@kth.se (M.S.T.)

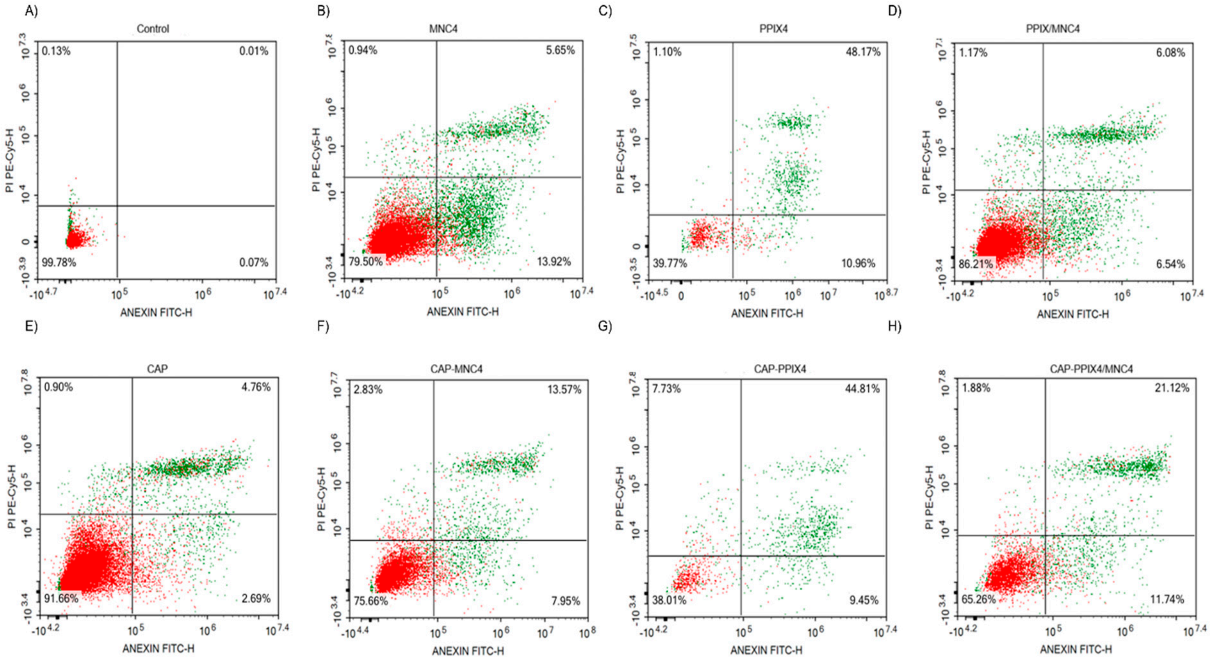

Figure S1. Representative Annexin V-FITC/PI flow cytometry plots of HaCaT cells under dark conditions: (A) Control, (B) MNC4, (C) PPIX4, (D) PPIX4@MNC4, (E) CAP, (F) CAP-MNC4, (G) CAP-PPIX4, and (H) CAP-PPIX4@MNC4. Cell populations were classified as viable, early apoptotic, late apoptotic, or necrotic based on Annexin V-FITC and propidium iodide staining after exclusion of debris and doublets using FSC/SSC and singlet gating.

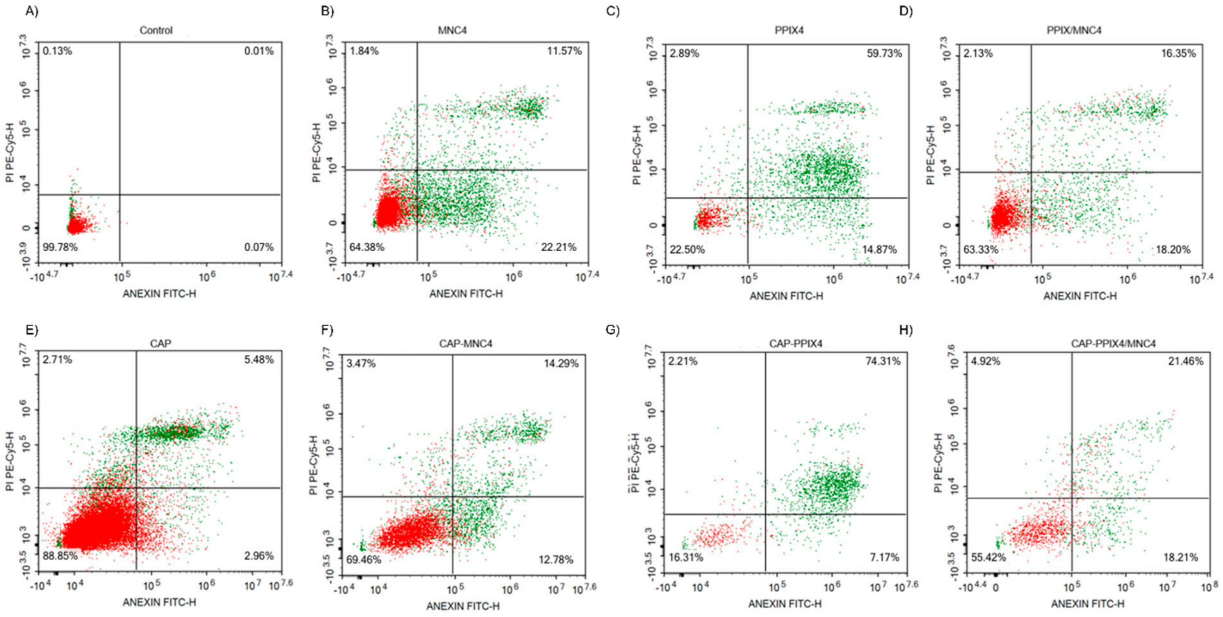

Figure S2. Representative Annexin V-FITC/PI flow cytometry plots of HaCaT cells under light conditions: (A) Control, (B) MNC4, (C) PPIX4, (D) PPIX4@MNC4, (E) CAP, (F) CAP-MNC4, (G) CAP-PPIX4, and (H) CAP-PPIX4@MNC4. Cell populations were classified as viable, early apoptotic, late apoptotic, or necrotic based on Annexin V-FITC and propidium iodide staining after exclusion of debris and doublets using FSC/SSC and singlet gating.

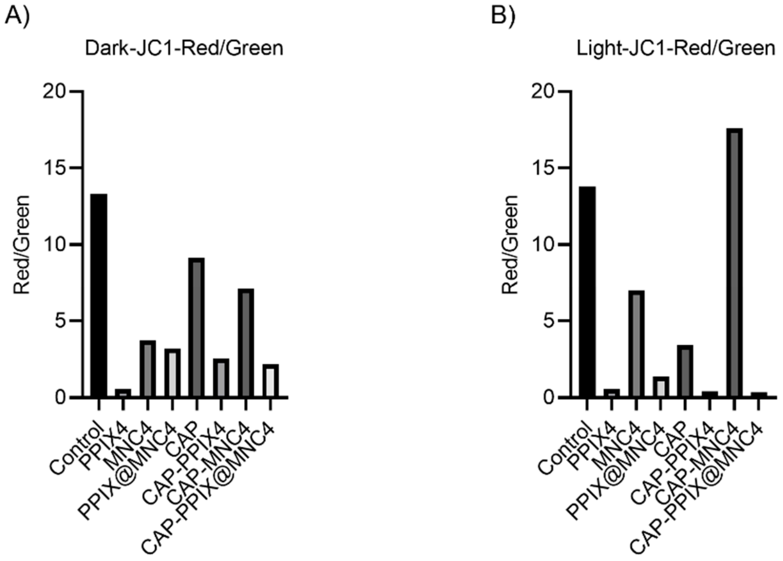

Figure S3. Population-based JC-1 red/green mitochondrial membrane potential ratio was calculated as the ratio of polarized mitochondria (Q2-2) to depolarized mitochondria (Q2-4). (A) Dark-treated groups. (B) Light-treated groups.
